# When a non-inflatable implant breaks the penis: A case report of penile fracture due to an unusual penile implant

**DOI:** 10.1016/j.ijscr.2024.109420

**Published:** 2024-02-20

**Authors:** Yufi Aulia Azmi, Johan Renaldo, Tri Budiyanto, Karinda Triharyu Caesari Putri, Hajid Rahmadianto Mardihusodo

**Affiliations:** a Departement Urology, Soetomo General Academic Hospital, Faculty of Medicine Universitas Airlangga, Surabaya, Indonesia; b Department of Health Sciences, University of Groningen, University Medical Center Groningen, Groningen, The Netherlands; c Departement Urology, Prof. Dr. Margono Soekarjo General Hospital, Purwokerto, Indonesia; d Faculty of Medicine, Universitas Jenderal Soedirman, Purwokerto, Indonesia

**Keywords:** Penile fracture, Implant, Toothbrush, Surgical

## Abstract

**Introduction and importance:**

Penile fracture is a relatively uncommon condition that most commonly results from blunt trauma during sexual intercourse, forced flexion, masturbation, or rolling over. However, other causes are also possible. It is a catastrophic illness to the patient's organic and psychological health.

**Case presentation:**

We report a case of a 43-year-old male patient who sustained a penile fracture due to the presence of a toothbrush implant. No urethral injury was documented. The patient underwent surgical repair, and one month post-surgery, exhibited no deformity and had a normal sexual and voiding function.

**Clinical discussion:**

Penile fracture is most commonly caused by blunt trauma during sexual intercourse. While it has not been documented in the literature**,** penile implants may increase the incidence of penile fractures. Clinical examination and urethrography confirmed the absence of urethral injury. In cases where a penile fracture is suspected, the only management is surgical exploration. This approach has resulted in the lowest rate of negative long-term sequelae and does not negatively impact the patient's psychological well-being.

**Conclusion:**

Penile fracture is a rare but serious condition that can result from the presence of an unstandardized implant. It is not close to the incidence of penile fracture for unstandardized prosthesis as even the standardized implant can get fracture when counter to high velocity. To avoid functional and morphological abnormalities, surgical exploration is recommended as the primary course of treatment.

## Highlights

• Penile fracture is a condition that results from the rupture of the tunica albuginea
• The ecchymotic and enlarged phallus often deviates to the side opposing the tunical tear
• Surgical repair can still be effective if performed within 7 days of injury.

## 1 Introduction and importance

Penile fracture is a rare but potentially serious condition that results from the rupture of the tunica albuginea of the penile corpora due to trauma to the erect penis. In the United States, it occurs at a frequency of just 1 in 175,000 hospital admissions and most commonly affects sexually active males between the ages of 30 and 50 [1]. However, the true incidence of this condition is unknown. Various studies estimate the incidence between 0.29 and 3.1 cases per 100,000 population [2]. In Indonesia, several case reports have focused on clinical manifestations rather than etiology [3]. The most common causes of penile fracture are sexual intercourse, forced flexion, masturbation, and rolling over, accounting for 46 %, 21 %, 18 %, and 8.2 % of cases, respectively. Other causes have also been reported [4]. Genital beading using tools like toothbrushes is an endemic and traditional practice in Indonesia. This toothbrush implant makes the penis look more structured to please their sexual partners. However, the application is not risk-free and exposes the risk of penile fracture. In this case report, we present an unusual instance of a penile fracture patient managed in a governmental hospital resulting from the presence of a toothbrush implant. This work has been reported in accordance with the SCARE and PROCESS criteria [5].

## 2 Case presentation

**Fig. 1:**
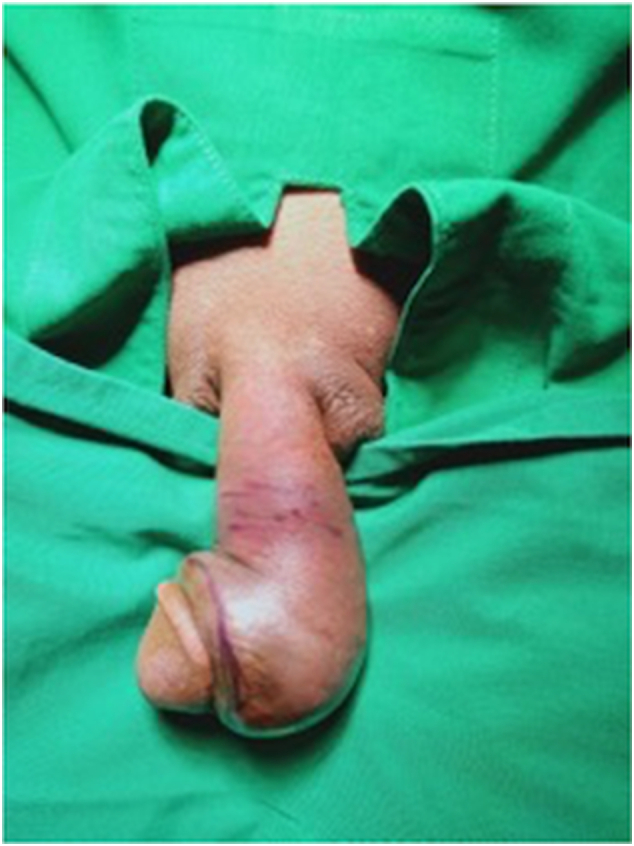
The clinical picture of penile fracture.

**Fig. 2:**
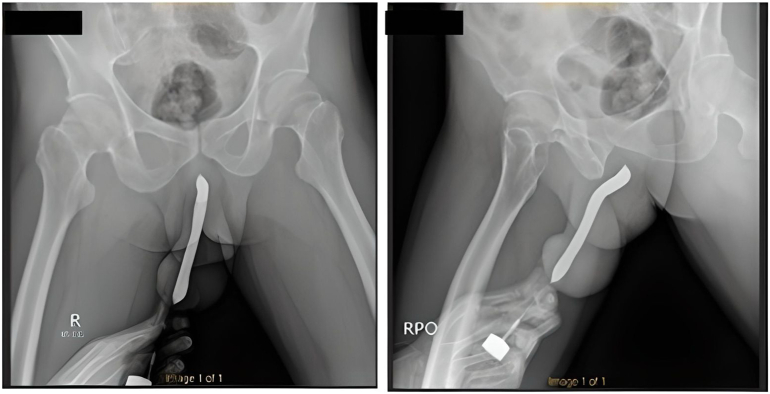
The urethral patency was confirmed by urethrography.

**Fig. 3:**
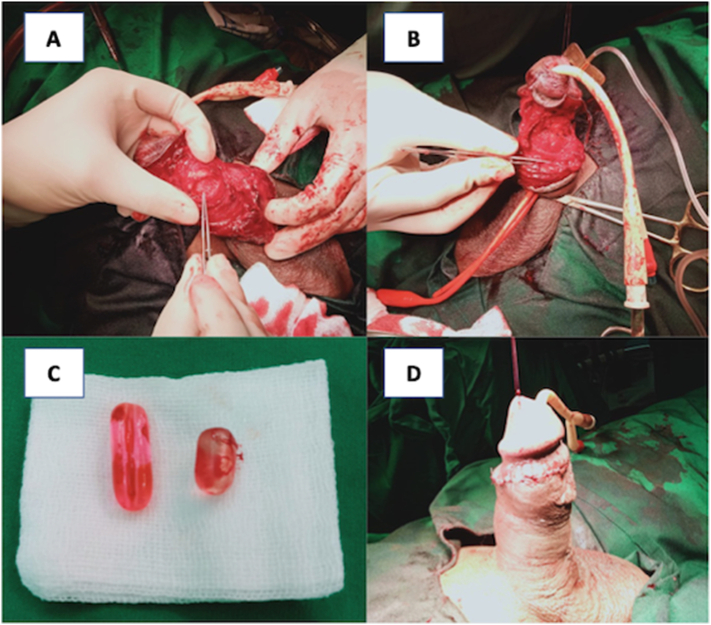
(A-B) Corpus repair; (C) Toothbrush implant; (D) After repair.

**Fig. 4:**
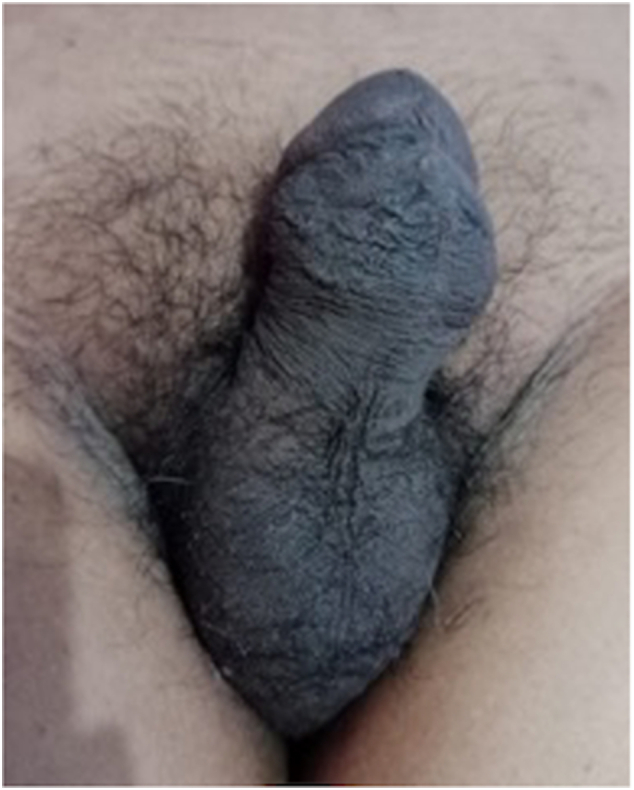
One month after surgical repair.

We present a case of penile fracture with concomitant toothbrush implants in a 43-year-old male patient who presented to our emergency department at a governmental hospital in East Java, Indonesia. The patient sustained a penile injury during sexual intercourse in the woman-on-top position the night before. Times since the accident until he came to the emergency ward, around 12 h. He reported penile curvature, pain, and swelling. He had a history of self-insertion of toothbrushes on the dorsal and ventral of his penis through a small incision at his home in 2017 for sexual satisfaction. Therefore, the patient had these tools in his penis for six years. On physical examination, he was alert and hemodynamically stable. He had an “eggplant deformity” of the penis with a palpable mass measuring 0.8 cm on the dorsal side and 1 cm on the ventral side (Fig. 1). There was no history of urethral injury, such as blood at the meatus or haematuria and no urinary retention. The laboratory tests were within normal limits. He received intravenous analgesics and anti-inflammatory agents. A retrograde urethrography confirmed the patency of the urethra (Fig. 2.). The patient underwent penile exploration under spinal anesthesia. During operative management, we found the left corpus cavernosa**,** which was torn 2 cm**,** and hematoma. We used a tourniquet to minimize bleeding. We did hematoma evacuation, and once the hematoma was successfully evacuated, we removed the toothbrush implant, and attention was turned to repairing tunica albuginea of corpus cavernosum to restore structural integrity and function to the penis (Fig. 3.). The difficulty of this surgery is to free the toothbrush implant adhesions from the surrounding tissue. A urologist with expertise in trauma and reconstruction performed the surgery. The total blood loss volume was ±100–150 cc, and the length time of the operation was two hours. The patient was discharged three days after surgery, and postoperative recovery proceeded without complications. At the one-month follow-up, the patient reported an absence of deformity and normal sexual and voiding function, and the Erection Hardness Scale (EHS) was 4 (Fig. 4). Overall, the patient was satisfied with the result.

## 3 Discussion

The thickness of the tunica albuginea, which is around 2 mm thick in the flaccid condition, drops to 0.25–0.5 mm during erection, increasing its susceptibility to severe damage [6]. Usually, during intense sexual activity, the stiff penis falls out of the vagina and hits the pubic bone or perineum, causing a buckled injury [1]. The presence of a penile implant may raise the chance of penile fracture because the implant as a foreign body leads to inflammation and results in the formation of a fibrous scar. This process reduces the focal fiber elasticity of the penis, which theoretically increases the risk of penile fracture. This hasn't been demonstrated in the literature. Additionally, it has been demonstrated that rolling over or falling upon an erect penis, masturbating, and other situations may result in penile fractures [7]. The tunica albuginea's remarkable tensile strength allows it to resist rupture up to intracavernous pressures of more than 1500 mmHg. A transverse laceration of the proximal penile shaft occurs when the erect penis curves inappropriately because the sudden rise in intracavernosal pressure surpasses the tensile strength of the tunica albuginea. Although most fractures occur distal to the suspensory ligament, this rupture, typically 1 to 2 cm in length, may occur anywhere along the penile shaft [8,9]. Because the tunica albuginea is thinnest in these areas, injuries related to coitus are often ventral or lateral [6,9].

When a patient's medical history and physical examination are combined, it is often possible to make an accurate diagnosis of a penile fracture. An abrupt cracking or popping sound, discomfort, rapid detumescence, and localized edema are the symptoms of this illness. Buck's penile hematoma will be trapped between the skin and tunica if his fascia is unbroken, giving rise to the recognizable “eggplant deformity.” On the other hand, if Buck's fascia is damaged, the hematoma could spread to other locations, such as the scrotum, perineum, and suprapubic areas. Buck's fascia was intact since the subject in our case study had an eggplant deformity. Because of the mass effect and hematoma, the ecchymotic and enlarged phallus frequently deviates to the side opposite the tunical tear [1]. In our case, the phallus deviated dorsally, indicating a ventral tunical tear. The implant was inserted in ventral and dorsal positions, with longer dorsal sides.

Preoperative urethrography is advised in situations of suspected urethral damage in penile fracture patients. It has been claimed that damage to the tunica albuginea may be accurately and noninvasively shown using magnetic resonance imaging (MRI) [10]. However, MRI is not often employed in the examination of patients with symptoms and physical findings indicative of penile fracture owing to considerations including cost, restricted availability, and time constraints. False fractures and vascular trauma are examples of differential diagnoses for penile fractures. Patients presenting with symptoms like penile edema and ecchymosis have been documented to have false fractures; some have even described the typical “snap-pop” or fast detumescence related to fracture [11]. Another disease that may manifest similarly to a penile fracture is a rupture of the dorsal penile artery or vein while in sexual intercourse [12].

It is advised that patients who are suspected of having a penile fracture seek immediate surgical examination and treatment. This entails shutting the tunica albuginea, which has been shown to have the lowest incidence of unfavorable long-term consequences and no detrimental effects on the patient's mental health [13]. To fully deglove the penis, the surgical technique usually entails creating a circular incision close to the coronal sulcus. On the other hand, ventral longitudinal approaches or small longitudinal incisions focused on the fracture site have become more common [14]. Before making an incision, a flexible cystoscopy may be carried out if urethral trauma is suspected of helping with localization [15,18].. After surgery, the tunica needs to be stitched with absorbable sutures, and patients should refrain from sexual activity for one month and get medication with broad-spectrum antibiotics. Reconstruction surgery has been linked to shorter recovery periods, fewer complications, lower rates of morbidity, and a lower prevalence of permanent penile curvature [16,17,19]. In our case study, the patient presented to the emergency room immediately following the incident. In cases where presentation is delayed, surgical repair can still be effective if performed within seven days of injury. However, the impact of the timing of surgical repair on long-term outcomes in patients with penile fractures remains a subject of debate [20].

Research has shown that up to 20 % of those with a history of penile fracture may have post-operative problems. With rates of 13.9 %, 2.8 %, and 1.9 %, respectively, these problems may include erectile dysfunction, the creation of plaques or nodules, and information of post-operative curvature [4]. A higher incidence of sequelae, including penile abscess, penile curvature, undetected urethral rupture, and persistent hematoma necessitating delayed surgical intervention, has been linked to conservative therapy of penile fractures [21]. Moreover, it has been observed that impotence may occur in as many as 62 % of patients and that fibrosis and angulations occur in 35 % of patients after conservative care [20,22]. It has been shown that the lowest incidence of detrimental long-term effects on the patient's functional and psychological well-being occurs from surgical treatment of penile fractures. Because of this, patients must understand the advantages of surgery while treating penile fractures. One month after surgery, the patient came to follow up with no morphological or functional problems from his report, and the Erection of Hardness Scale (EHS) was 4, which means the penis was completely hard and had fully rigid erections. We did not perform any imaging modality to assess postoperative fibrosis owing to the high cost and lack of imaging facility.

## 4 Conclusion

Penile fracture is an uncommon but serious medical condition that might result from unusual instances, including the presence of foreign objects such as a toothbrush implant. The current standard of care for this condition is surgical exploration. Current recommendations generally advise patients to undergo immediate repair and avoid using unstandardized implants. Further research is essential to refine treatment guidelines, thereby enhancing the overall quality of clinical practice in this rare but significant condition.

Inform Consent.

Written inform consent were acquired from patient.

## Ethical approval

Ethical approval for this study was provided by Health Research Ethics Committee of Dr. Soetomo General-Academic Hospital, Surabaya.

## Sources of funding

This research did not receive any specific grant from funding agencies in the public, commercial, or not-for-profit sectors.

## Author contributions

Yufi Aulia Azmi: Conceptualization, Methodology, Data Curation, Investigation, Writing-Original draft preparation.

Hajid Rahmadianto: Conceptualization, Data Curation, Writing-Original draft preparation.

Karinda Triharyu Caesari Putri: Writing-Original draft preparation.

Tri Budiyanto: Writing-Original draft preparation.

Johan Renaldo: Supervision, Validation, Writing-Reviewing, and Editing.

## Guarantor

Johan Renaldo

## Conflicts of interest

The authors declare that there is no conflict of interest.
